# Working conditions and mental health of employees suffering from common mental disorders in Germany with special consideration to the migration generation

**DOI:** 10.1186/s40359-026-05486-2

**Published:** 2026-09-01

**Authors:** Regina Herold, Yesim Erim, Ute B. Schröder, Meike Heming, Eva Rothermund-Nassir, Jeannette Weber, Nicole Rosalinde Hander, Eva Morawa

**Affiliations:** 1 Department of Psychosomatic Medicine and Psychotherapy, University Hospital of Erlangen, Friedrich-Alexander University Erlangen-Nürnberg (FAU), Erlangen, Germany; 2 Federal Institute for Occupational Safety and Health, Division “Work and Health”, Berlin, Germany; 3 Institute of Occupational, Social, and Environmental Medicine, Centre for Health and Society, Medical Faculty, University Hospital Düsseldorf, Heinrich Heine University Düsseldorf, Düsseldorf, Germany; 4 Department of Psychosomatic Medicine and Psychotherapy, Ulm University Medical Center, Ulm, Germany

**Keywords:** Working conditions, Workplace, Migrants, Migration background, Mental health, Occupational health

## Abstract

**Purpose:**

This study examines working conditions, mental health and their association among mentally burdened employees in Germany attending psychotherapeutic consultation at work, considering first- and second-generation migrants.

**Methods:**

Baseline data from a randomized controlled trial on psychotherapeutic consultation at work (friaa study) were analyzed. Using χ²- and two-sided *t*-tests and one-way ANOVAs, differences between individuals with and without migration background (MB) as well as individuals without MB and first- and second-generation migrants were assessed regarding working conditions (self-constructed questions, five scales of the COPSOQ, PSC) and depressive (PHQ-9), anxiety (GAD-2) and somatic symptoms (SSS-8). Associations between working conditions and mental health were calculated for the entire sample and separately for individuals with and without MB using linear regression analyses.

**Results:**

Among 549 participants (54.8% female, mean age: 45.9 years (*SD* = 11.0), 12.4% had a MB. Individuals with MB more often worked in jobs associated with lower socio-economic status (blue-collar work: *p*=.008, fewer subordinates: *p*<.001, less possibilities for development: *p*=.004). Poorer work environment (especially in individuals without MB) (all βs≥0.124, all *p*s≤0.008) and quantitative job demands (especially in individuals with MB) (all βs≥0.118, all *p*s≤0.071, partially only statistical trends) were associated with worse mental health. Higher possibilities for development (in individuals with and without MB) (all βs≥-0.102, all *p*s≤0.071), social support (all βs≥-0.111, all *p*s≤0.016) and psychosocial safety climate (all βs≥-0.104, all *p*s≤0.044) (both especially in individuals without MB) were related to better mental health.

**Conclusion:**

Findings suggest the importance of improving and tailoring working conditions to employees‘ needs, including consideration of MB.

**Trial registration number:**

Registered at the German Clinical Trial Register (DRKS) at 01.03.2021 (DRKS00023049).

## Introduction

Poor working conditions constitute a potential risk factor for mental health issues [1–4]. The Job-Demand-Control-Support model by Karasek [5] and the Job-Demands-Ressources Model by Bakker and Demerouti [6] highlight that a balance between job demands such as physical strain and quantitative pressures and resources is essential for maintaining good mental health. In line with this, high work resources improve coping strategies and job satisfaction as well as mental health [5, 6]. Numerous studies confirm that reducing demands and strengthening resources supports well-being [7, 8]. Accordingly, possibilities for development (skill discretion), influence at work (decision authority), and leadership quality were found to be key protective factors of mental health and therefore, work ability [9].

In 2022, 15% of the global workforce were affected by mental disorders [4]. In the general adult population in Germany, the prevalence rates appear to be even higher, with a representative study showing that 28% of adults experienced a mental disorder within a one-year period [10]. In addition, the Institute for Workplace Health Promotion reported that approximately 40% of company decision-makers identified mental health symptoms as a major issue in the workplace [11]. Mental illness accounts for 18% of all work incapacity days in Germany, causing substantial economic costs [12]. Therefore, identifying work-related and socio-demographic risk factors for mental health is of high relevance for labor and health policy.

One of those socio-demographic risk factors can be found in the migration status. Over the past decades, migration to Europe has steadily increased [13], with Germany being the main destination of migration (Bundesamt für Migration und Flüchtlinge [14]). These migration movements are driven by various factors, including forced migration due to war and persecution, which contributed to the major refugee influxes between 2014 and 2016 [15] and in 2022 [16]. In addition, labour and education have become increasingly important drivers of migration to Germany (Bundesamt für Migration und Flüchtlinge [17]). In 2025, approximately 26.0 million persons in Germany had a migration background (MB), representing 31% of the total population. Also among the employed population in Germany, 31% had a MB (Statistisches Bundesamt [18]), highlighting the growing importance of this population group within the German labour market. Migrants more often face unfavorable working conditions such as low-skilled jobs, overqualification, discrimination, and poorer psychosocial working environments compared to individuals without MB [1, 19, 20]. This can often be explained by overqualification due to non-recognition of foreign qualifications [1, 2, 21]. These factors are negatively associated with mental well-being [1, 2, 22]. Psychosocial safety climate, reflecting the extent to which organizations prioritize and support employees’ mental health [23], may be particularly relevant in this context. Previous research has highlighted its relevance for refugee workers [24]. These already mentioned less favorable psychosocial working conditions may also be associated with lower perceived psychosocial safety climate among persons with MB. Given their increased vulnerability in terms of mental issues [19, 25], it is essential to examine work-related risks in this population. According to the Microcensus, a regular household survey conducted in Germany by the Federal Statistical Office, individuals with MB can be devided as follows. First-generation migrants are defined as individuals who do not have German citizenship by birth. Second-generation migrants are defined as individiuals born in Germany of whom at least one parent does not have German citizenship by birth [26]. These groups may differ in working conditions and mental health. First-generation migrants often experience long-term disadvantages, while second-generation migrants tend to align more with the native population in terms of working conditions [27]. Due to inconsistent findings regarding mental well-being across migration generations, it remains unclear which generation faces greater challenges [28–31]. In order to specifically identify potentially differing factors that are associated with poorer mental health or poorer working conditions in the respective groups, these two migration populations must be analyzed separately.

The present study focuses on mentally burdened employees who were interested in visiting psychotherapeutic consultation at work. This group is particularly relevant as they are at an increased risk of work-related health problems but are also willing to change. In this context, working conditions are key factors influencing mental health [1–4]. The differentiation according to MB and migration generation takes place against the background of work-related inequalities [1, 21, 22] which can also have an impact on the experience of distress [1, 2] and therefore, the need for psychotherapeutic intervention. The aim is to identify differentiated risk profiles and starting points for culturally sensitive prevention.

For this reason, it was examined whether mentally burdened employees with and without MB as well as without MB and first- and second-generation migrants differ in terms of

1. psychosocial working conditions (employment extent, weekly working hours and days, (night) shift work, occupational group, management role, number of subordinates, company size, quantitative job demands, influence at work (decision authority), possibilites for development (skill discretion), social support, work environment (physical demands), psychosocial safety climate),
2. mental health (depressive, anxiety, somatic symptoms, International Classification of Diseases (ICD-10) diagnoses), and
3. the association between working conditions and mental health (controlled for age, gender, and marital status, and conducted separately for individuals with and without MB).

## Methods

### Setting and population

Baseline data from the „friaa“ project („Frühe Intervention am Arbeitsplatz“), a longitudinal multicenter randomized controlled trial designed to evaluate the effectiveness of early psychotherapeutic intervention for psychologically burdened employees before the onset of clinical disorders, were used. Five centers in Germany (Ulm, Düsseldorf, Teltow, Hildesheim, Erlangen) recruited participants from 09/2021 to 01/2023 through various channels such as company physicians, multipliers, social media, and newspapers. Eligible participants were ≥ 18 years old, worked ≥ 15 h/week, had sufficient German skills, and either had an ICD-10 diagnosis [32] or subclinical psychosomatic symptoms (Global Assessment by Functioning Scale, GAF < 81) [33]. Baseline data were collected before intervention and randomization to intervention or control group. Both the intervention group and the control group were included in this study. Further study details and information on the intervention are described in Weber et al., [34], the results regarding the effectiveness of the intervention can be found in Rothermund-Nassir et al., [35].

### Measures

All questionnaires were self-assessment instruments and were completed by the subjects themselves.

#### Sociodemographic data

The questionnaire included socio-demographic characteristics such as age, gender, marital status and MB and was presented in German. According to Destatis [26], first-generation migrants were defined as individuals who negated the following question: „Have you had German citizenship since birth?“. Second-generation migrants were defined as individuals who affirmed the question about German citizenship since birth, but negated the following question: „Have your father and mother had German citizenship since birth?“.

#### Work-related conditions

Data was collected on the extent of work (full-time vs. part-time), the number of weekly working hours and days, (night) shift work, the occupational group, holding a management position and if so, the amount of employees one is responsible for, and the company size.

The following subscales of the third validated German version of the Copenhagen Psychosocial Questionnaire (COPSOQ) were taken into account: 1) „Quantitative job demands“, 2) „Influence at work (decision authority)“, 3) „Possibilities for development (skill discretion)“, 4) „Social support“ (by colleagues and supervisors) and 5) „Work environment (physical demands)“. Answers were recoreded to a five-point scale from 100 („always”/„to a very large extent“) to 0 („never/almost never”/„to a very small extent“) [9]. A higher value indicates a higher level of the corresponding item or subscale [36]. Individual item scores as well as the mean scores of each subscale are reported. In the present sample, the reliability for the subscales „Quantitative job demands“ and „Social Support“ (both Cronbach’s α = 0.80) could be interpreted as good, for „Possibilities for development (skill discretion)“ as acceptable (Cronbach’s α = 0.75) and for „Influence at work (decision authority)“ (Cronbach’s α = 0.62) and „Work environment (physical demands)“ (Cronbach’s α = 0.69) as questionable.

Furthermore, the German version of the Psychosocial Safety Climate Scale-4 (PSC-4) was applied [37]. It was answered on a five-point scale from 1 („strongly disagree”) to 5 („strongly agree”). The total sum score ranges from 4 to 20. A higher value indicates a better psychosocial safety climate [23]. Both individual item scores and a global sum score are presented. In the present sample, the internal consistency can be described as good (Cronbach’s α = 0.85).

#### Mental health

The Patient Health Questionnaire-9 (PHQ-9) was used in the validated German version to record depressive symptoms. Answers were given on a four-point scale from 0 („not at all“) to 3 („almost every day“). Clinically relevant depressive symptoms can be assumed from a sum value of ≥ 10 (value range from 0 to 27) [38]. With Cronbach’s α = 0.80, the reliability can be assessed as good in the present sample.

Symptoms of generalized anxiety were assessed using the validated German version of the Generalized Anxiety Disorder Scale-2 (GAD-2). It was answered on a scale from 0 („not at all“) to 3 („almost every day“). A sum score of ≥ 3 indicates clinically relevant anxiety symptoms (value range from 0 to 6) [39]. In the present sample, the internal consistency can be interpreted as acceptable (Cronbach’s α = 0.75).

Using the validated German version of the Somatic Symptom Scale-8 (SSS-8), somatic symptoms were measured. Answers were given on a scale from 0 („not at all“) to 4 („very much“). A sum value of ≥ 12 indicates clinically relevant somatic symptoms (value range from 0 to 32) [40]. In the present sample, the reliability proved to be acceptable with Cronbach’s α = 0.74.

The MINI International Neuropsychiatric Interview (German Translation Version 6.0.0) [41] was conducted by psychotherapists to obtain clinical diagnoses of common mental disorders according to the ICD-10 [32].

### Statistical analyses

Statistical analyses were calculated using IBM SPSS Statistics 29.0.1.0. Missing values were imputed using the Expectation Maximization Method (COPSOQ, PSC-4, SSS-8). Relative and absolute frequencies and sum and mean values (*M*) and their standard deviations (*SD*) were reported to characterize socio-demographic, migration-, work- and health-related data. Both mean and cut-off values derived from the original questionnaires were used to describe depressive, anxiety and somatic symptoms. Furthermore, ICD-diagnoses are presented. For clarity, depressive and manic/bipolar disorders were categorized under „Affective disorders“ (F3) and post-traumatic stress, anxiety, obsessive-compulsive, adjustment, somatoform and other neurotic disorders under „Other neurotic, stress and somatoform disorders“ (F4). Furthermore, the categories „Behavioral disorders with physical disorders and factors“ (F5), „Personality disorders“ (F6) and „Hyperkinetic disorders“ (F9) are presented. Exploratory group comparisons between persons with and without MB as well as between persons without MB, first-generation and second-generation migrants were performed using two-sided *t*-tests and one-way ANOVAs with bonferroni-adjusted post-hoc tests for continuous variables, and χ²-tests with bonferroni-adjusted post-hoc *Z*-tests for categorial variables. Effect sizes were reported using ϕ, Cramer’s *V* and *p*η²: |0.1| ≤ ϕ/Cramer’s *V* < |0.3| = small, |0.3| ≤ ϕ/Cramer’s *V* < |0.5| = moderate, ϕ/Cramer’s *V* ≥ |0.5| = large effect size; *p*η² = |0.010| ≤ *p*η² < |0.039| = small, |0.060| ≤ *p*η² < |0.140| = moderate, *p*η² ≥ |0.140| = large effect size [42].

The following assumptions for conducting regression analyses were checked: outliers, normal distribution of residuals using the histogram and the P-P plot, linearity and homoscedasticity using the scatter plot, autocorrelation using the Durbin-Watson test (1.77–2.03), and multicollinearity using the variance inflation factor (VIF) (1.04-2.00). In order to avoid multicollinearity, bivariate Pearson-correlations were calculated (data not shown). No coefficients exceeded *r* = 0.7, so all variables were retained. Multivariable linear regression analyses were then conducted to investigate the association between working conditions and mental health outcomes (depressive, anxiety, and somatic symptoms), controlling for key socio-demographic data (age, gender: male vs. female, marital status: in a partnership vs. single, and MB: yes vs. no). Work-related factors included extent of work (full- vs. part-time), mean scores of the COPSOQ subscales „Quantitative job demands“, „Influence at work (decision authority)“, „Possibilities for development (skill discretion)“, „Social support“, „Work environment (physical demands)“ and the PSC-4 sum score. Analyses were calculated for the whole sample and for individuals with and without MB separately. Individuals of diverse gender or with unclear employment status (e.g., trainees, unpaid work, or non-specified categories) were excluded from the regression analyses (*n* = 10).

For all analyses, an alpha error level of α = 0.05 (two-sided) was used to test statistical significance.

## Results

In the following section, only significant differences are described. Significant and non-significant effects can be found in the corresponding tables.

### Sample

The sample consisted of 550 participants. One person was excluded from all analyses due to too many missings (*n* = 549).

### Socio-demographic characteristics

Information on socio-demographic characteristics can be found in Table 1. Among the participants, 12.4% (*n* = 68) reported having a MB. Of these, 52.9% (*n* = 36) were first-generation migrants and 47.1% (*n* = 32) were second-generation migrants. Specific migration-related information of this sample is available in a previously published article [43]. In the total sample, 54.8% were female (*n* = 301). The mean age was 45.89 years (*SD* = 11.04). Almost two thirds of the participants were in a partnership or married (60.5%, *n* = 332).

**Table 1:** Socio-demographic and work-related data

	Total (*n* = 549, 100%) (A)	Individuals without MB (*n* = 481, 87.6%)(B)	Individuals with MB (*n* = 68, 12.4%)(C)	Difference between B and C, *p*-value (effect size^1^)(D)	First-generation migrants (*n* = 36, 52.9%)(E)	Second-generation migrants (*n* = 32, 47.1%)(F)	Difference between B, E and F^1^, *p*-value (effect size^2^)(G)
**Gender,** ***n*** **(%)**				0.352 (0.062)			0.560 (0.052)
Female	301 (54.8)	269 (55.9)	32 (47.1)		15 (42.9)	15 (46.9)	
Male	247 (45.0)	211 (43.9)	36 (52.9)		21 (60.0)	17 (53.1)	
Diverse sex	1 (0.2)	1 (0.2)	0 (0.0)		0 (0.0)	0 (0.0)	
**Age,** ***M*** **(** ***SD*** **)**	45.89 (11.04)	**46.40 (10.97)** ^3^_**a**_^4^	**42.31 (10.96)**	**0.004 (0.373)**	43.53 (10.72)_a, b_	**40.94 (11.23)**_**b**_	**0.010 (0.017)**
Min-Max	21-64	21-64	21-62				
Missing, %	1 (0.2)	1 (0.2)	0 (0.0)				
**Marital status,** ***n*** **(%)**				0.668 (0.053)			0.593 (0.065)
Married/permanent partnership	332 (60.5)	289 (60.1)	43 (63.2)		25 (69.4)	18 (56.3)	
Single	144 (26.2)	127 (26.4)	17 (25.0)		9 (25.0)	8 (25.0)	
Divorced	63 (11.5)	55 (11.4)	8 (11.8)		2 (4.6)	6 (18.8)	
Widowed	10 (1.8)	10 (2.1)	0 (0.0)		0 (0.0)	0 (0.0)	
**Employment,** ***n*** **(%)**				0.353 (0.090)			0.418 (0.086)
Full-time	402 (73.2)	347 (72.1)	55 (80.9)		31 (86.1)	24 (75.0)	
Part-time	138 (25.1)	126 (26.2)	12 (17.7)		5 (13.9)	7 (21.9)	
Other^5^	9 (1.6)	8 (1.7)	1 (1.5)		0 (0.0)	0 (0.0)	
**Weekly working hours,** ***M*** **(** ***SD*** **)**	36.89 (7.14)	36.77 (7.11)	37.77 (7.36)	0.282 (0.141)	38.17 (8.82)	37.32 (5.28)	0.499 (0.003)
Missing, %	3 (0.6)	2 (0.4)	1 (1.5)		0 (0.0)	0 (0.0)	
**Weekly working days,** ***M*** **(** ***SD*** **)**	4.92 (0.58)	4.90 (0.58)	5.03 (0.55)	0.085 (0.223)	5.14 (0.59)	4.91 (0.47)	0.058 (0.010)
**Shift work,** ***n*** **(%)**				0.627 (0.041)			0.777 (0.040)
No shift work	475 (86.5)	418 (86.9)	57 (83.8)		29 (80.6)	28 (87.5)	
Shift work with night shifts	40 (7.3)	35 (7.3)	5 (7.4)		3 (8.3)	2 (6.3)	
Shift work without night shifts	34 (6.2)	28 (5.8)	6 (8.8)		4 (11.1)	2 (6.3)	
**Occupational groups,** ***n*** **(%)**				**0.008 (0.114)**			**0.008 (0.132)**
Blue Collar Workers	131 (23.9)	**106 (22.0)**_**a**_	**25 (36.8)**		**16 (44.4)**_**b**_	9 (28.1)_a, b_	
White Collar Workers	418 (76.1)	**375 (78.0)**_**a**_	**43 (63.2)**		**20 (55.6)**_**b**_	23 (71.9)_a, b_	
**Leadership position,** ***n*** **(%)**				0.088 (0.073)			0.135 (0.085)
No	433 (78.9)	374 (77.8)	59 (88.8)		33 (91.7)	26 (81.3)	
Yes	116 (21.1)	107 (22.2)	9 (13.2)		3 (8.3)	6 (18.8)	
**Number of subordinate employees,** ***M*** **(SD)** ^6^	24.60 (40.38)	**25.98 (41.73)**	**8.22 (6.10)**	**< 0.001 (0.441)**	5.00 (5.00)	9.83 (6.34)	0.445 (0.014)
**Company size,** ***n*** **(%)**				**0.011 (0.165)**			0.130 (0.120)
Large (> 1000 employees)	339 (61.7)	295 (61.3)	44 (64.7)		23 (63.9)	21 (65.6)	
Large, smaller number of employees (250 – 1000 employees)	100 (18.2)	**96 (20.0)**_**a**_	**4 (5.9)**_**b**_		**1 (2.8)**_**b**_	3 (9.4)_a, b_	
Medium-sized (50 – 249 employees)	60 (10.9)	**46 (9.6)**_**a**_	**14 (20.6)**_**b**_		**8 (22.2)**_**b**_	6 (18.8)_a, b_	
Small (10 – 49 employees)	38 (6.9)	33 (6.9)	5 (7.4)		4 (11.1)	1 (3.1)	
Very small (2 – 9 employees)	10 (1.8)	10 (2.1)	0 (0.0)		0 (0.0)	0 (0.0)	
One man	1 (0.2)	1 (0.2)	0 (0.0)		0 (0.0)	0 (0.0)	
Missing	1 (0.2)	0 (0.0)	1 (1.5)		0 (0.0)	1 (3.1)	

^1^χ²-tests with Bonferroni-adjusted α for categorial variables, *t*-tests for independent samples or one-factor ANOVAs for continuos variables; ^2^ϕ and Cramer’s *V*s for χ²-tests, Cohen’s *d*s for t-tests or *p*η² for ANOVAs; ^3^Significant differences are marked in bold; ^4^Subscript letters refer to the comparison between persons without migration background, first- and second-generation migrants, deviating letters indicate a significant difference between the respective groups, identical letters indicate no difference (calculated using post-hoc *t*-tests with Bonferroni-adjusted α); ^5^This category includes trainees, voluntarily (unpaid) employment, not applicable; ^6^Question only concerns those individuals who stated that they hold a management position

### Work-related characteristics

Further work-related characteristics of this sample, which are not taken into account in the analyses in this study, can be found in previously published articles [43–45]. Details on work-related conditions considered in the present study are provided in Table 1.

#### Self-constructed questions

Almost three-quarters worked full-time (73.2%, *n* = 402). The average number of working hours per week was 36.89 h (*SD* = 7.14). The average number of working days per week was 4.92 days (*SD* = 0.58). The vast majority did not work shifts (86.5%, *n* = 475). Among those who reported shift work (13.5%, *n* = 74), 54.1% (*n* = 40) worked night shifts. White collar workers were the most common occupational group (76.1%, *n* = 418). Individuals with MB (*n* = 25, 36.8%) significantly more often worked as blue collar workers than individuals without MB (*n* = 106, 22.0%) (χ²(1) = 7.113, *p* = 0.008, Cramer’s *V* = 0.114). This is mainly due to the difference between individuals without MB and first-generation migrants (*n* = 16, 44.4%) (χ²(2) = 9.596, *p* = 0.008, Cramer’s *V* = 0.132). A total of 21.1% (*n* = 116) of participants stated holding a management position. Managers were responsible for an average of 24.60 employees (*SD* = 40.38). Individuals with MB (*M* = 8.22, *SD* = 6.10) were responsible for significantly less subordinates than individuals without MB (*M* = 25.98, *SD* = 41.73) (*t*(89.878) = 3.931, *p* < 0.001, Cohen’s *d* = 0.441). Most respondents worked in a large company of more than 1000 employees (61.7%, *n* = 339). Individuals without MB more often worked in a large company with 250 to 1000 employees (20.0%, *n* = 96) than individuals with MB (5.9%, *n* = 4) (χ²(5) = 14.945, *p* = 0.011, Cramer’s *V* = 0.165). This can be explained by the significant difference between individuals without MB and first-generation migrants (2.8%, *n* = 1). Furthermore, individuals without MB (9.6%, *n* = 46) were less likely to work in medium-sized companies (50 to 249 employees) than individuals with MB (20.6%, *n* = 14). This difference can be explained by the significant difference between individuals without MB and first-generation migrants as well (22.2%, *n* = 8) (χ²(10) = 17.039, *p* = 0.074, Cramer’s *V* = 0.125).

#### Psychosocial working conditions (COPSOQ) and psychosocial safety climate (PSC-4)

Results on the scores of the COPSOQ and the Psychosocial Safety Climate (PSC-4) can be found in Table 2. With regard to the mean scores of the subscales, individuals without MB had more possibilities for development (higher skill discretion) at work (*M* = 64.1, *SD* = 21.3) than first-generation migrants (*M* = 53.2, *SD* = 25.7) (post-hoc *t*-test: *t*(515) = 2.896, *p* = 0.004, Cohen’s *d* = 0.500, bonferroni-adjusted α of 0.017).

**Table 2:** Average total scores of the subscales and the individual items of the Copenhagen Psychosocial Questionnaire (COPSOQ) and the Psychosocial Safety Climate Scale (PSC-4)

	**Total** (*n* = 549, 100%)(A)	**Individuals without MB** (*n* = 481, 87.6%)(B)	**Individuals with MB** (*n* = 68, 12.4%)(C)	**Difference between B and C**^1^**, p-value (effect size**^2^**)**(D)	**First-generation migrants** (*n* = 36, 52.9%)(E)	**Second-generation migrants** (*n* = 32, 47.1%)(F)	**Difference between B, E and F**^1^**, p-value (effect size**^2^**)**(G)
**Quantitative job demands** ^3^ **,** ***M*** **(** ***SD*** **)**							
Total mean score	55.3 (21.4)	55.5 (21.2)	53.4 (22.9)	0.448 (0.098)	56.8 (25.4)	49.6 (19.4)	0.289 (0.005)
*Item 1:* Do you have to work very quickly?	55.9 (26.2)	56.1 (26.1)	54.0 (26.8)	0.538 (0.080)	57.6 (27.3)	50.0 (26.2)	0.403 (0.003)
*Item 2:* Do you work at high speed all day?	53.1 (27.7)	53.3 (27.9)	52.2 (25.8)	0.771 (0.038)	54.9 (28.5)	49.2 (22.4)	0.675 (0.001)
*Item 3:* How often do you find that you don''t have enough time to complete all your tasks?	61.6 (28.9)	62.1 (28.4)	58.2 (32.4)	0.306 (0.133)	61.8 (36.6)	54.2 (27.0)	0.332 (0.004)
*Item 4:* Are you falling behind with your work?	54.4 (29.7)	54.4 (29.8)	54.4 (29.3)	0.998 (0.000)	59.7 (31.8)	48.4 (25.4)	0.296 (0.004)
*Item 5:* Do you have to work overtime?	51.1 (30.7)	51.6 (30.6)	48.2 (31.5)	0.404 (0.108)	50.0 (33.8)	46.2 (29.2)	0.623 (0.002)
**Influence at work (decision authority)** ^3^ **,** ***M*** **(** ***SD*** **)**							
Total mean score	40.2 (23.0)	40.0 (22.8)	41.7 (25.0)	0.558 (0.076)	37.5 (27.2)	46.5 (21.6)	0.233 (0.005)
*Item 1:* Do you have much influence on decisions that affect your work?	47.9 (27.2)	48.2 (27.2)	45.6 (27.0)	0.458 (0.096)	43.1 (29.0)	48.4 (24.5)	0.545 (0.002)
*Item 2:* Do you have any influence on the amount of work you are given?	32.5 (26.9)	**31.7 (26.6)** ^4^_**a**_^5^	37.9 (28.5)	0.077 (0.230)	31.9 (30.8)_a,b_	**44.5 (24.4)**_**b**_	**0.032 (0.013)**
**Possibilities for development (skill discretion)** ^3^ **,** ***M*** **(** ***SD*** **)**							
Total mean score	63.5 (21.5)	**64.1 (21.3)**_**a**_	59.1 (22.0)	0.071 (.234)	**53.2 (25.7)**_**b**_	65.6 (14.6)_a,b_	**0.011 (0.016)**
*Item 1:* Does your work offer variety?	66.7 (25.9)	**67.5 (25.8)**_**a**_	61.4 (26.1)	0.068 (.237)	**53.5 (28.8)**_**b**_	**70.3 (19.5)**_**a**_	**0.005 (0.019)**
*Item 2:* Do you have the opportunity to learn new things through your work?	55.5 (27.4)	55.8 (27.4)	53.3 (27.3)	0.486 (.090)	50.7 (28.3)	56.3 (26.2)	0.554 (0.002)
*Item 3:* Can you apply your skills or expertise to your work?	67.9 (25.7)	**68.6 (25.6)**_**a**_	62.4 (26.1)	0.066 (0.239)	**55.6 (30.5)**_**b**_	**70.3 (17.3)**_**a**_	**0.011 (0.016)**
**Social support** ^3^ **,** ***M*** **(** ***SD*** **)**							
Total mean score	59.1 (22.5)	58.9 (22.4)	60.6 (23.1)	0.552 (0.077)	59.3 (26.6)	62.0 (18.7)	0.744 (0.001)
*Item 1:* How often do you receive help and support from your colleagues when needed?	64.2 (25.8)	64.4 (25.7)	63.4 (27.4)	0.781 (0.036)	62.5 (30.2)	64.5 (24.4)	0.914 (0.000)
*Item 2:* How often are your colleagues willing to listen to your work problems if necessary?	64.7 (26.2)	65.1 (26.3)	62.3 (25.7)	0.420 (0.105)	60.4 (29.5)	64.5 (20.8)	0.587 (0.002)
*Item 3:* How often do you receive help and support from your immediate supervisor when needed?	49.7 (30.7)	48.8 (30.8)	56.1 (29.2)	0.067 (0.237)	57.4 (31.1)	54.7 (27.3)	0.176 (0.006)
*Item 4:* How often is your immediate supervisor willing to listen to your work problems if necessary?	58.1 (30.7)	57.7 (31.2)	60.5 (26.4)	0.434 (0.090)	57.1 (29.1)	64.3 (22.7)	0.493 (0.003)
**Work environment (physical demands)** ^3^ **,** ***M*** **(** ***SD*** **)**							
Total mean score	23.4 (22.2)	23.4 (22.3)	23.3 (22.3)	0.989 (0.002)	26.9 (23.3)	19.3 (20.8)	0.366 (0.004)
*Item 1:* How often do you have to do heavy physical work, e.g. heavy lifting, carrying or lifting?	18.1 (28.2)	17.5 (28.0)	22.4 (29.7)	0.179 (0.174)	25.0 (32.2)	19.5 (26.7)	0.295 (0.004)
*Item 2:* How often are you exposed to noise or loud ambient noises at your workplace?	41.2 (34.3)	41.2 (34.3)	41.8 (34.5)	0.885 (0.019)	46.3 (34.0)	36.7 (34.8)	0.509 (0.002)
*Item 3:* How often do you come into contact with chemicals or hazardous substances at work?	13.2 (26.1)	13.2 (26.5)	12.9 (23.1)	0.918 (0.013)	17.4 (27.3)	7.8 (16.1)	0.320 (0.004)
*Item 4:* How often are you exposed to draughts or extreme temperatures at your workplace?	28.6 (30.6)	28.7 (30.2)	28.3 (33.7)	0.928 (0.012)	33.3 (36.8)	22.7 (29.3)	0.356 (0.004)
*Item 5:* How often are you exposed to bad air at work, e.g. cigarette smoke, gases or similar?	16.3 (26.8)	16.4 (26.9)	15.8 (25.9)	0.865 (0.022)	19.4 (29.3)	11.7 (21.0)	0.488 (0.003)
*Item 6:* How often are you exposed to poor lighting conditions at work, e.g. bright or dim light?	22.5 (28.0)	23.1 (28.4)	18.8 (24.6)	0.234 (0.154)	20.1 (26.6)	17.2 (22.4)	0.448 (0.003)
**Psychosocial Safety Climate** ^6^ **,** ***M*** **(SD)**							
Total sum score	10.30 (3.78)	10.27 (3.77)	10.49 (3.84)	0.643 (.060)	10.15 (3.71)	10.88 (3.77)	0.651 (0.002)
*Item 1*: The management is committed to avoiding stress and is involved in this.	2.53 (1.09)	2.51 (1.08)	2.66 (1.11)	0.266 (0.144)	2.63 (1.02)	2.70 (1.22)	0.516 (0.002)
*Item 2*: The management considers the mental health of employees to be just as important as their performance for the company.	2.77 (1.16)	2.76 (1.17)	2.88 (1.10)	0.429 (0.103)	2.77 (1.04)	2.99 (1.16)	0.538 (0.002)
*Item 3*: Our company often addresses the topic of mental health.	2.57 (1.21)	2.58 (1.22)	2.49 (1.16)	0.537 (0.080)	2.43 (1.23)	2.55 (1.10)	0.765 (0.001)
*Item 4*: All levels in our company have the opportunity to help prevent stress.	2.43 (1.08)	2.42 (1.08)	2.47 (1.05)	0.732 (0.044)	2.32 (1.01)	2.64 (1.09)	0.446 (0.003)

^1^One-factor ANOVAs or *t*-tests for independent samples with Bonferroni-adjusted α; ^2^*p*η² for ANOVAs or Cohen’s *d*s for *t*-tests; ^3^COPSOQ-subscale; ^4^Significant differences are marked in bold; ^5^Subscript letters refer to the comparison between individuals without MB, first- and second-generation migrants, deviating letters indicate a significant difference between the respective groups, identical letters indicate no difference (calculated using post-hoc *t*-tests with Bonferroni-adjusted α); ^6^PSC-4

Regarding the mean score of the individual items of the subscale „Influence at work (decision authority)“, individuals without MB (*M* = 31.7, *SD* = 26.6) reported a significantly lower influence on the amount of work assigned to them than second-generation migrants (*M* = 44.5, *SD* = 24.4) (post-hoc *t*-test: *t*(510) = -2.655, *p* = 0.008, Cohen’s *d* = 0.485; bonferroni-adjusted α of 0.017). The mean score of the individual items of the subscale „Possibilities for development (skill discretion)” showed that first-generation migrants‘ (*M* = 53.5, *SD* = 38.8) work varies significantly less than the work of individuals without MB (*M* = 67.5, *SD* = 25. 8) (post-hoc *t*-test: *t*(514) = 3.125, *p* < 0.001, Cohen’s *d* = 0.540, bonferroni-adjusted α = .017) and second-generation migrants (*M* = 70.3, *SD* = 19.5) (post-hoc *t*-test: *t*(61.892) = -2.852, *p* = 0.003, bonferroni-adjusted α = 0.017). In addition, first-generation migrants (*M* = 55.6, *SD* = 30.5) can significantly less often apply their skills or expertise in their work than individuals without MB (*M* = 68.6, *SD* = 25.6) (post-hoc *t*-test: *t*(514) = 2.916, *p* = 0.002, Cohen’s *d* = 0.504, bonferroni-adjusted α = 0.017) and second-generation migrants (*M* = 70.3, *SD* = 17.3) (post-hoc *t*-test: *t*(56.550) = -2.485, *p* = 0.008, Cohen’s *d* = 0.585, bonferroni-adjusted α = 0.017).

### Mental health

An overview of all mental health outcomes is shown in Table 3.

**Table 3:** Mental health outcomes and ICD-10 diagnoses (World Health Organization, 2004)

	Total (*n* = 549, 100%)(A)	Individuals without MB (*n* = 481, 87.6%)(B)	Individuals with MB (*n* = 68, 12.4%)(C)	Difference between B and C^1^, *p*-value (effect size^2^)(D)	First-generation migrants (*n* = 36, 53.9%)(E)	Second-generation migrants (*n* = 32, 46.2%)(F)	Difference between B, E and F^3^, *p*-value (effect size^4^)(G)
**Depressive symptoms,** ***M*** **(** ***SD*** **)**	12.91 (5.12)	12.80 (5.03)	13.69 (5.72)	0.178 (0.175)	14.22 (6.01)	13.09 (5.40)	0.268 (0.005)
**Frequency of clinically relevant depressive symptoms** ^5^ **,** ***n*** **(%)**				0.288 (0.045)			0.555 (0.046)
Not clinically relevant Clinically relevant	142 (25.9) 407 (74.1)	128 (26.6) 353 (73.4)	14 (20.6) 54 (79.4)		7 (19.4) 29 (80.6)	7 (21.9) 25 (78.1)	
**Generalized anxiety symptoms,** ***M*** **(** ***SD*** **)**	3.46 (1.71)	3.43 (1.72)	3.65 (1.60)	0.323 (0.128)	3.72 (1.63)	3.56 (1.58)	0.570 (0.002)
**Frequency of clinically relevant generalized anxiety symptoms** ^5^ **,** ***n*** **(%)**				0.490 (0.029)			0.712 (0.035)
Not clinically relevant Clinically relevant	190 (34.6) 359 (65.4)	169 (35.1) 312 (64.9)	21 (30.9) 47 (69.1)		12 (33.3) 24 (66.7)	9 (28.1) 23 (71.9)	
**Somatic symptoms,** ***M*** **(** ***SD*** **)**	13.17 (5.72)	13.19 (5.61)	13.04 (6.46)	0.848 (0.025)	14.17 (6.88)	11.78 (5.80)	0.225 (0.005)
**Frequency of clinically relevant somatic symptoms** ^5^ **,** ***n*** **(%)**				0.468 (0.031)			0.603 (0.043)
Not clinically relevant Clinically relevant	228 (41.5) 321 (58.5)	197 (41.0) 284 (59.0)	31 (45.6) 37 (54.4)		15 (41.7) 21 (58.3)	16 (50.0) 16 (50.0)	
**Diagnose,** ***n*** **(%)**				0.317 (0.104)			0.774 (0.077)
No diagnosis F3 – Affective disorders^6^ F4 - Other neurotic, stress-related and somatoform disorders^7^ F5 – Behavioral disorders associated with physical factors F6 – Personality disorders F9 – Hyperkinetic disorders Missing	84 (15.3) 285 (51.9) 161 (29.3) 11 (2.0) 1 (0.2) 5 (0.9) 2 (0.4)	77 (16.0) 244 (50.5) 144 (29.9) 8 (1.7) 1 (0.2) 5 (1.0) 2 (0.4)	7 (10.3) 41 (60.3) 17 (25.0) 3 (4.4) 0 (0.0) 0 (0.0) 0 (0.0)		4 (11.1) 21 (58.3) 9 (25.0) 2 (0.5.6) 0 (0.0) 0 (0.0) 0 (0.0)	3 (9.4) 20 (62.5) 8 (25.0) 1 (3.1) 0 (0.0) 0 (0.0) 0 (0.0)	

^1^χ²-tests with Bonferroni-adjusted α for categorial variables, *t*-tests for independent samples; ^2^ϕ and Cramer’s *V*s for χ²-tests, Cohen’s *d*s for *t*-tests; ^3^χ²-tests with Bonferroni-adjusted α for categorial variables or one-factor ANOVAs for continuos variables; ^4^ϕ and Cramer’s *V*s for χ²-tests, Cohen’s *d*s for t-tests or *p*η² for ANOVAs; ^5^Using the cut-offs (Depressive symptoms, PHQ-9: Sum score ≥ 10; Generalized anxiety symptoms, GAD-2: Sum score ≥ 3; Somatic symptoms, SSS-8: Sum score ≥ 12); ^6^contains depressive disorder, manic/bipolar disorder; ^7^contains post-traumatic stress disorder, anxiety disorder, obsessive-compulsive disorder, adjustment disorder, somatoform disorder, other neurotic disorder

#### Depressive symptoms

The mean score for depressive symptoms for the entire sample was 12.91 (*SD* = 5.12). Almost three-quarters (74.1%, *n* = 407) of all participants showed clinically relevant depressive symptoms (PHQ-9 ≥ 10).

#### Generalized anxiety symptoms

The mean anxiety score of the total sample was 3.46 (*SD* = 1.71). Almost two thirds (65.4%, *n* = 359) showed clinically relevant generalized anxiety values (GAD-2 ≥ 3).

#### Somatic symptoms

The mean somatic symptom score of the total sample was 13.17 (*SD* = 5.72). More than half of the participants (58.5%, *n* = 321) suffered from clinically relevant somatic symptoms (SSS-8 ≥ 12).

#### Clinical diagnoses

A clinical diagnosis according to the ICD-10 was exhibited by 84.3% (*n* = 463). Affective disorders (F3) were the most frequently represented diagnosis group with 51.9% (*n* = 285), followed by neurotic stress and somatoform disorders (F4) with 29.3% (*n* = 161).

### Association between work-related characteristics and mental health in the total sample

Results on the multivariable linear regression models of the total sample can be found in Table 4.

**Table 4:** Multivariable linear regression analyses of the total sample

Independent variables	Model 1: Depressive symptoms (PHQ-9) (*R*² = 6.9%, *R*²_adj_ = 4.9%				Model 2: Generalized anxiety symptoms (GAD-2) (*R*² = 5.6%, *R*²_adj_ = 3.6%)				Model 3: Somatic symptoms (SSS-8) (*R*² = 12.2%, *R*²_adj_ = 10.4%)			
	Regression coefficient (95% CI)	SE	β	*p*	Regression coefficient (95% CI)	SE	β	*p*	Regression coefficient (95% CI)	SE	β	*p*
Constant	14.014	1.726	-	**< 0.001** ^1^	2.864	0.580	-	**< 0.001**	8.674	1.880	-	**<0.001**
Gender (female vs. male)^2^	0.920	0.465	0.090	**0.049**	0.301	0.157	0.088	0.055	1.332	0.507	0.116	**0.009**
Age	-0.009	0.021	-0.020	0.647	-0.001	0.007	-0.005	0.917	0.055	0.022	0.102	**0.015**
MB (yes vs. no)^3^	0.643	0.661	0.042	0.331	0.217	0.222	0.042	0.330	0.194	0.721	0.011	0.788
Partnership (yes vs. no)^4^	0.204	0.455	0.019	0.654	0.217	0.153	0.062	0.156	0.522	0.495	0.047	0.265
Employment situation (part-time vs. full-time)^5^	-0.623	0.523	-0.053	0.234	0.199	0.176	0.051	0.258	0.822	0.570	0.063	0.149
COPSOQ: „Quantitative job demands“	0.017	0.011	0.072	0.120	0.010	0.004	0.127	**0.007**	0.018	0.012	0.068	0.130
COPSOQ: „Influence at work (decision authority)“	0.004	0.011	0.020	0.691	0.001	0.004	0.016	0.752	0.003	0.012	0.012	0.804
COPSOQ: „Possibilities for development (skill discretion)“	-0.039	0.012	-0.165	**0.001**	-0.009	0.004	-0.112	**0.032**	-0.039	0.013	-0.146	**0.004**
COPSOQ: „Social support“	-0.005	0.011	-0.022	0.633	0.003	0.004	0.039	0.411	-0.028	0.012	-0.111	**0.016**
COPSOQ: „Work environment (physical demands)“	0.030	0.010	0.130	**0.003**	0.001	0.003	0.018	0.685	0.055	0.011	0.214	**< 0.001**
PSC-4 sum score	-0.058	0.064	-0.043	0.364	-0.051	0.022	-0.113	**0.028**	0.027	0.070	0.018	0.700

^1^Significant factors are marked in bold; ^2^Ref: male; ^3^Ref: no MB; ^4^Ref: no partnership; ^5^Ref: full-time

#### Depressive symptoms

A worse „work environment (higher physical demands)“ was significantly associated with higher depressive symptoms (β = 0.130, *p* = 0.003). Good „possibilities for development (high skill discretion)“ were significantly related to lower levels of depressive symptoms (β = − 0.165, *p* = 0.001).

#### Generalized anxiety symptoms

High „quantitative job demands“ showed a significant association with higher generalized anxiety symptoms (β = 0.127, *p* = 0.007), while good „possibilities for development (high skill descretion)“ (β = − 0.112, *p* = 0.032) and a high „psychosocial safety climate“ (β = − 0.113, *p* = 0.028) were significantly related to lower levels of generalized anxiety.

#### Somatic symptoms

A poor „work environment (high physical demands)“ was significantly associated with higher somatic symptoms (β = 0.214, *p* < 0.001). Good „possibilities for development (high skill descretion)“ (β = − 0.143, *p* = 0.004) and receiving „social support“ (β = − 0.111, *p* = 0.016) were significantly related to less somatic symptoms.

### Assocation between work-related characteristics and mental health among individuals without MB vs. individuals with MB

Results on the multivariable linear regression models of individuals with vs. without MB are presented in Table 5.

**Table 5:** iAUCs for blood plasma GLP1 concentrations in the first 60, 120 and overall 180 min in response to treatments

Individuals without MB														
Independent variables	Model 1: Depressive symptoms (PHQ-9) (*R*² = 5.5%, R²_adj_ = 3.4%)				Model 2: Generalized anxiety symptoms (GAD-2) (*R*² = 5.2%, *R*²_adj_ = 3.2%)				Model 3: Somatic symptoms (SSS-8) (*R*² = 11.1%, *R*²_adj_ = 9.1%)					
	Regression coefficient (95% CI)	SE	β	*p*	Regression coefficient (95% CI)	SE	β	*p*	Regression coefficient (95% CI)	SE		β		*p*
Constant	13.451 (9.830-17.073)	1.843	-	**<0.001** ^1^	2.550 (1.305-3.795)	0.634	-	**<.001**	8.655 (4.699-12.611)	2.013		-		**<0.001**
Gender (female vs. male)^2^	0.921 (-0.050-1.893)	0.494	0.092	0.063	0.322 (-0.011-0.656)	0.170	0.093	0.058	1.061 (-0.001-2.122)	0.540		0.094		0.050
Age	-0.005 (-0.047-0.038)	0.022	-0.010	0.830	0.003 (-0.011-0.018)	0.007	0.020	0.664	0.060 (0.013-0.107)	0.024		0.114		**0.012**
Partnership (yes vs. no)^3^	0.360 (-0.582-1.302)	0.479	0.035	0.453	0.238 (-0.086-0.562)	0.165	0.067	0.150	0.541 (-0.488-1.570)	0.524		0.047		0.302
Employment situation (part-time vs. full-time)^4^	-0.518 (-1.594-0.558)	0.548	-0.046	0.344	0.229 (-0.141-0.599)	0.118	0.059	0.224	0.970 (-0.206-2.145)	0.598		0.076		0.106
COPSOQ: „Quantitative job demands“	0.012 (-0.011-0.035)	0.012	0.051	0.306	0.010 (0.002-0.018)	0.004	0.118	**0.019**	0.009 (-0.016-0.035)	0.013		0.035		0.476
COPSOQ: „Influence at work (decision authority)“	0.003 (-0.020-0.026)	0.012	0.013	0.804	0.001 (-0.006-0.009)	0.004	0.019	0.722	0.005 (-0.020-0.030)	0.013		0.019		0.716
COPSOQ: „Possibilities for development (skill discretion)“	-0.034 (-0.059--0.009)	0.013	-0.146	**0.008**	-0.008 (-0.017-0.000)	0.004	-0.102	0.063	-0.027 (-0.054-0.001)	0.014		-0.102		0.055
COPSOQ: „Social support“	-0.004 (-0.026-0.019)	0.011	-0.017	0.742	0.003 (-0.005-0.010)	0.004	0.036	0.485	-0.036 (-0.060--0.011)	0.012		-0.142		**0.004**
COPSOQ: „Work environment (physical demands)“	0.028 (0.007-0.049)	0.011	0.124	**0.008**	0.001 (-0.006-0.008)	0.004	0.016	0.740	0.053 (0.030-0.076)	0.012		0.209		**<0.001**
PSC-4 sum score	-0.048 (-0.182-0.085)	0.068	-0.036	0.478	-0.047 (-0.093--0.001)	0.023	-0.104	**0.044**	0.044 (-0.101-0.190)	0.074		0.030		0.551

**Individuals with MB**														
**Independent variables**	**Model 1: Depressive symptoms (PHQ-9)** **(** ***R*** **² = 20.4%,** ***R*** **²**_**adj**_ **= 6.2%)**				**Model 2: Generalized anxiety symptoms (GAD-2)** **(** ***R*** **² = 15.5%,** ***R*** **²**_**adj**_ **= 0.4%)**				**Model 3: Somatic symptoms (SSS-8)** **(** ***R*** **² = 41.6%,** ***R*** **²**_**adj**_ **= 31.2%)**					
	Regression coefficient (95% CI)	SE	β	*p*	Regression coefficient (95% CI)	SE	β	*p*	Regression coefficient (95% CI)		SE		β	*p*
Constant	19.116 (8.981-29.250)	5.069	-	**<0.001** ^1^	5.274 (2.354-8.194)	1.458	-	**<0.001**	7.718 (-1.918-17.345)		4.810		-	0.114
Gender (female vs. male)^2^	0.736	1.528	0.065	0.632	0.013 (-0.869-0.895)	0.440	0.004	0.977	4.205 (1.293-7.116)		1.453		0.332	0.005
Age	-0.066 (-0.205-0.073)	0.069	-0.124	0.345	-0.035 (-0.075-0.005)	0.020	-0.233	0.087	0.003 (-0.130-0.135)		0.066		0.004	0.969
Partnership (yes vs. no)^3^	-1.272 (-4.511-1.967)	1.617	-0.107	0.435	-0.067 (-1.000-.866)	0.466	-0.020	0.886	-0.238 (-3.318-2.841)		1.537		-0.018	0.877
Employment situation (part-time vs. full-time)^4^	-1.604 (-5.305-2.096)	1.847	-0.108	0.389	-0.042 (-1.108-1.024)	0.532	-0.010	0.937	0.203 (-3.315-3.722)		1.756		0.012	**0.908**
COPSOQ: „Quantitative job demands“	0.070 (-0.006-0.145)	0.038	0.270	0.069	0.020 (-0.002-0.042)	0.011	0.276	0.071	0.109 (0.037-0.181)		0.036		0.379	**0.004**
COPSOQ: „Influence at work (decision authority)“	0.005 (-0.067-0.076)	0.036	0.020	0.896	-0.002 (-0.022-0.019)	0.010	-0.027	0.867	0.016 (-0.052-0.084)		0.034		0.063	0.636
COPSOQ: „Possibilities for development (skill discretion)“	-0.081 (-0.169-0.007)	0.044	-0.311	0.071	-0.014 (-0.040-0.011)	0.013	-0.199	0.258	-0.173 (-0.256--0.089)		0.042		-0.597	**<0.001**
COPSOQ: „Social support“	-0.005 (-0.073-0.062)	0.034	-0.022	0.871	0.005 (-0.015-0.024)	0.010	0.070	0.619	0.041 (-0.023-0.106)		0.032		0.151	0.202
COPSOQ: „Work environment (physical demands)“	0.034 (-0.032-0.100)	0.033	0.134	0.302	0.003 (-0.016-0.022)	0.009	0.041	0.757	0.047 (-0.016-0.110)		0.031		0.165	0.138
PSC-4 sum score	-0.073 (-0.492-0.346)	0.209	-0.049	0.727	-0.059 (-0.179-0.062)	0.060	-0.139	0.335	-0.096 (-0.494-0.302)		0.199		-0.057	0.631

^1^Significant factors are marked in bold; ^2^Ref: male; ^3^Ref: no partnership; ^4^Ref: full-time

#### Depressive symptoms

Among individuals without MB, a poor „work environment (high physical job demands)“ was significantly associated with higher depressive symptoms (β = 0.124, *p* = 0.008) and „possibilities for development (skill discretion)“ were significantly related to lower depressive symptoms (β = − 0.146, *p* = 0.008). For individuals with MB, statistical trends of associations between „quantitative job demands“ and higher depressive symptoms (β = 0.270, *p* = 0.069) as well as „possibilities for development (skill descretion)“ and lower depressive symptoms (β = − 0.311, *p* = 0.071) were found.

#### Generalized anxiety symptoms

For individuals without MB, high „quantitative job demands“ showed a significant association with higher anxiety symptoms (β = 0.118, *p* = 0.019), a high „psychosocial safety climate“ was significantly associated with lower anxiety symptoms (β = − 0.104, *p* = 0.044) and good „possibilites for development (high skill discretion)“ showed a statistical trend of an association with lower anxiety symptoms (β = − 0.102, *p* = 0.063). Among individuals with MB, „quantitative job demands“ showed a statistical trend of an association with higher anxiety symptoms (β = 0.276, *p* = 0.071).

#### Somatic symptoms

Among individuals without MB, a worse „work environment (high physical demands)“ was significantly correlated to higher somatic symptoms (β = 0.209, *p* < 0.001), „social support“ was significantly related to lower somatic symptoms (β = − 0.142, *p* = 0.004) and better „possibilities for development (higher skill discretion)“ showed a statistical trend of an association with lower somatic symptoms (β = − 0.102, *p* = 0.055). For individuals with MB, „quantitative job demands“ were significantly associated with higher somatic symptoms (β = 0.379, *p* = 0.004) and „possibilities for development (skill discretion)“ were significantly correlated to lower somatic symptoms (β = − 0.597, *p* < 0.001).

## Discussion

The present study examined the differences in working conditions and mental health between individuals with and without MB in Germany in mentally burdened employees. Special consideration was given to the differentiation between first- and second-generation migrants. Furthermore, the association between working conditions and mental health was examined in the whole sample as well as separately among individuals with and without MB. 

### Working conditions

Exploratory analyses showed that individuals with MB were more often employed in blue-collar jobs, particularly those of the first generation. This can be attributed to overqualification, which affects many migrants, especially those of the first generation, due to the non-recognition of foreign qualifications [1, 2, 21]. Since certified credentials are often required for higher-level jobs in countries like Germany, individuals with MB are forced to seek low-skilled employment with lower socio-economic status [1, 19, 21]. Such disadvantages may persist even after years of residence, as barriers encountered during the initial labor market integration might have lasting effects on career trajectories and opportunities for occupational advancement. Among managers, those without MB supervised significantly more employees than those with MB. On the one hand, this may be explained by the fact that individuals with MB more often worked in medium-sized companies, while those without MB were more likely to be employed in larger companies with greater leadership responsibilities. Another factor could be their younger average age (42 vs. 46 years), possibly indicating less professional experience and fewer opportunities for senior management roles. On the other hand, the lower level of responsibility among individuals with MB could suggest structural discrimination, such as being perceived as less suited for leading larger teams. However, their equal representation in management positions challenges this assumption within the present sample. It should be noted that the first-generation migrants in this study had lived in Germany for an average of 24 years and were well integrated socially, linguistically, and economically. Previous findings in the friaa cohort showed good German language skills, strong orientation toward the host culture, low migration-related distress [43], and working conditions comparable to those without MB. Nevertheless, a high level of integration does not necessarily eliminate occupational disadvantages that originated during early stages of labor market integration and may continue to influence occupational attainment over time. Given their high level of integration, these individuals were not representative of all migrants in Germany, especially not of more recently arrived first-generation migrants, for whom structural discrimination may be even more pronounced. In terms of the extent of employment (full- vs. part-time), weekly working hours and days, shift work and leadership position no difference between individuals with and without MB and the migration generations could be found.

According to COPSOQ results, first-generation migrants reported significantly lower opportunities for development (poorer skill discretion) compared to those without MB, while individuals without MB and second-generation migrants did not differ significantly. This may be due to the non-recognition of foreign qualifications, leading to employment in lower-skilled jobs with limited development prospects [2, 19]. This is reflected in first-generation migrants‘ less frequent use of skills at work. In contrast, second-generation migrants, having received their education in Germany, experienced similar working conditions to those without MB. No significant group differences were found for other dimensions such as quantitative job demands, influence at work (decision authority), social support, work environment (physical demands), or psychosocial safety climate measured using the PCS-4. One possible explanation is that those work-related factors are primarily determined by job-specific conditions and are less dependent on the socio-cultural background of employees. In Germany, working conditions seem to be at least in parts standardized within comparable professions or working environments, meaning that individuals with and without MB are exposed to similar demands. This indicates that in terms of those working conditions, no systematic disadvantage or discrimination could be identified.

## Mental health

All three health outcomes (depressive, anxiety, and somatic symptoms) showed elevated, clinically relevant scores, reflecting the mentally burdened sample recruited for a psychotherapeutic intervention study. Therefore, the results are not representative of the general working population [43]. No significant differences were found between individuals with and without MB, or between individuals without MB and first- and second-generation migrants although previous studies highlighted the mental health vulnerability of migrants [19, 25]. The absence of group differences may be explained by the fact that the sample consisted of mentally burdened employees only. The vulnerability in terms of mental health among individuals with MB might only be shown in representative, i.e. healthy, samples, but not in subjects suffering from mental distress. Furthermore, it can be concluded that individuals with and without MB were apparently reached at the same stage of the disease. Another explanation might be protective factors such as high social support, mostly favorable working conditions, and strong social networks, supported by COPSOQ data showing generally positive perceptions of psychosocial work factors and strong social support at work, which might have compensated for the additional distressing factor of migration.

### Assocation between working conditions and mental health

In the entire sample, greater possibilities for development (skill descretion) were significantly linked to lower levels of all health symptoms. A poorer work environment (higher physical demands) was significantly associated with higher depressive and somatic symptoms. Social support was signifcantly correlated with fewer somatic symptoms, while higher quantitative job demands were significantly linked to increased anxiety symptoms. No association was found between influence at work (decision authority) and mental health. A positive psychosocial safety climate was significantly related to lower anxiety levels.

The results revealed significant associations between work-related factors and mental health, aligning with the job demand-control-support [5] and job demand-resources models [6]. In line with our findings, previous studies using the COPSOQ have also shown that greater possibilities for development (skill discretion) were associated with better mental health [46–48], whereas a poorer work environment (higher physical demands) was linked to reduced work ability [9]. Likewise, greater social support from supervisors has been associated with fewer psychological symptoms, including lower somatic distress [48]. Higher quantitative job demands have been related to increased burnout [7] as well as depression and anxiety disorders [49]. Finally, a low psychosocial safety climate has been associated with higher levels of psychological distress [50]. Surprisingly, influence at work (decision authority) showed no association with mental health, despite its central role in many models. This may be due to limitations in how influence was measured or to the stronger relevance of other factors like social support or work environment in this sample.

For individuals with MB, quantitative job demands were significantly linked to higher somatic symptoms, furthermore statistical trends of associations with higher depressive and anxiety symptoms were found. For those without MB, only a significant relationship with higher anxiety symptoms emerged. A worse work environment (higher physical demands) was significantly associated with higher depressive and somatic symptoms among individuals without MB. Among individuals with MB, no association with mental health was observed. Social support showed a significant association with lower somatic symptoms among individuals without MB. The psychosocial safety climate showed a significant correlation with lower anxiety levels among individuals without MB. Among individuals without MB, possibilities for development (skill discretion) showed a significant association with lower depressive symptoms and statistical trends of associations with lower anxiety and somatic symptoms, among individuals with MB, they significantly correlated with lower somatic symptoms and showed statistical trends of associations with lower depressive symptoms.

Quantitative job demands may have been associated with poorer mental health of individuals with MB due to cultural differences in distress perception and expectations regarding workload [51]. They may be less accustomed to Germany’s high work intensity, leading to greater strain. Among individuals with MB, no association was observed between poorer work environment (higher physical demands) and mental health, which could reflect greater resilience, possibly due to harsher working conditions in their countries of origin, where health protections may be less stringent. However, these assumptions are partially based on statistically non-significant trends, which must be further investigated in future research using larger sample sizes. Notably, social support was linked to better mental health among individuals without MB. This association was not found among individuals with MB, although both groups reported similar social support levels at work. One hypothesis to explain these results could lie in the cultural background of individuals with MB. As they might originate from collectivist countries [52], they may prioritize family networks over workplace support [53] and may be less likely to seek help from colleagues [54, 55]. Consequently, they might perceive available support as less effective or not utilize it. However, since countries of origin were not recorded, future studies should account for cultural background [2].

### Strengths and limitations

Strengths of the study include the recruitment across five different regions in Germany, reducing location-specific biases, and the comprehensive training of company doctors, which included intercultural competence and awareness of the needs of individuals with MB [34]. The use of the Microcensus definition for MB [26] enhances comparability with other migration studies in Germany. Furthermore, the wide range of work-related variables allowed for a multifaceted analysis [43].

One limitation of the study is the reliance on self-report questionnaires, which are susceptible to social desirability bias [56]. Furthermore, some of the validated questionnaires used showed lower internal consistency than in other previously published studies (e.g., Gierk [40], Kliem [57], Nübling [58], two subscales even were of questionable internal consistency. The cross-sectional design does not allow any conclusions to be drawn about causal relationships. (Very) low *R*² values in the regression analyses suggest that relevant health-related factors such as education, other specific working conditions, or migration-related variables (e.g. refugee status) may not have been considered in the present study. Future research should therefore consider a broader range of sociodemographic and migration-related factors. Further limitations include the small and unequal group sizes, potentially resulting in limited statistical power, especially given that the proposed maximum of one factor per ten participants was exceeded in this sample. This is reflected in large, yet non-significant regression coefficients (β weights), especially for individuals with MB. For this reason, these results should be interpreted with caution. Furthermore, it should be noticed that the present study does not involve moderator analyses in which MB was examined as a moderator of the relationship between working conditions and mental health. Due to the limited statistical power, this study merely examined the relationships between working conditions and mental health separately for individuals with and without MB. In future analyses with larger samples, interaction terms should be taken into account. The small sample of individuals with MB may stem from the general difficulty in recruiting individuals with MB („hard to reach population“ [59], who are often less willing to participate in scientific studies [60–62]. Statistically, this may have hindered the detection of small but meaningful effects. Additionally, individuals with MB were underrepresented (12% vs. 29% in the general workforce [63]). Future studies should apply the „community approach“ [64] and involve native-speaking team members to improve participation of individuals with MB. A further selection bias may have occurred due to the double pre-selection. Only employees from participating companies and those already perceiving a need for psychotherapeutic intervention were included. Therefore, the findings cannot be generalized to all employees in Germany which is confirmed by certain socio-demographic characteristics. While some characteristics aligned with national data (e.g., age, working hours), others did not (e.g., gender distribution, company size [43]. Despite these limitations, the study offers valuable exploratory insights for future research.

## Conclusion

Individuals with MB (particularly first-generation migrants) often face disadvantages in the workplace, including more frequent employment in blue-collar jobs, fewer leadership responsibilities, and limited opportunities for development (skill discretion). Regardless of MB, work-related factors such as possibilities for development (skill discretion), social support at work, and psychosocial safety climate are crucial for protecting mental health, while a poor work environment (high physical demands) as well as quantitative job demands may be linked to increased distress. These findings highlight the importance of improving work organization and psychosocial resources. Workplace interventions could include expanding access to training and career development, promoting supportive leadership or offering team building programs, strengthening psychosocial safety climate and reducing excessive quantitative and physical job demands (enhance the work environment), particularly in occupations where persons with MB are overrepresented.

## Data Availability

The datasets generated and/or analysed during the current study are not publicly available due to protection regulations.

## Abbrevations

ANOVA: Analysis of variance
CI: Confidence Interval
COPSOQ: Copenhagen Psychosocial Questionnaire
friaa: Frühe Intervention am Arbeitsplatz („Early intervention in the workplace“)
GAD-2: Generalized Anxiety Disorder Scale-2
GAF: Global Assessment by Functioning Scale
ICD-10: International Statistical Classification of Diseases-10
MB: Migration background
PSC-4: Psychosocial Safety Climate Scale-4
PHQ-9: Patient Health Questionnaire-9
SE: Standard Erro
SSS-8: Somatic Symptom Scale-8
VIF: Variance inflation factor
